# Implementing voluntary medical male circumcision using an innovative, integrated, health systems approach: experiences from 21 districts in Zimbabwe

**DOI:** 10.1080/16549716.2017.1414997

**Published:** 2018-01-11

**Authors:** Caryl Feldacker, Batsirai Makunike-Chikwinya, Marrianne Holec, Aaron F. Bochner, Abby Stepaniak, Robert Nyanga, Sinokuthemba Xaba, Peter H. Kilmarx, Amy Herman-Roloff, Taurayi Tafuma, Mufuta Tshimanga, Vuyelwa T. Sidile-Chitimbire, Scott Barnhart

**Affiliations:** ^a^ International Training and Education Center for Health (I-TECH), Seattle, WA, USA; ^b^ Department of Global Health, University of Washington, Seattle, WA, USA; ^c^ International Training and Education Center for Health (I-TECH), Harare, Zimbabwe; ^d^ Ministry of Health and Child Care, Harare, Zimbabwe; ^e^ U.S. Centers for Disease Control and Prevention, Harare, Zimbabwe; ^f^ Zimbabwe Community Health Intervention Project (ZiCHIRe), Harare, Zimbabwe; ^g^ Zimbabwe Association of Church-related Hospitals (ZACH), Harare, Zimbabwe; ^h^ Department of Medicine, University of Washington, Seattle, WA, USA

**Keywords:** Voluntary medical male circumcision, innovations in healthcare delivery, integrated service models, health system strengthening, Zimbabwe

## Abstract

**Background**: Despite increased support for voluntary medical male circumcision (VMMC) to reduce HIV incidence, current VMMC progress falls short. Slow progress in VMMC expansion may be partially attributed to emphasis on vertical (stand-alone) over more integrated implementation models that are more responsive to local needs. In 2013, the ZAZIC consortium began implementation of a 5-year, integrated VMMC program jointly with Ministry of Health and Child Care (MoHCC) in Zimbabwe.

**Objective**: To explore ZAZIC’s approach emphasizing existing healthcare workers and infrastructure, increasing program sustainability and resilience. Methods: A process evaluation utilizing routine quantitative data. Interviews with key MoHCC informants illuminate program strengths and weaknesses.

**Methods**: A process evaluation utilizing routine quantitative data. Interviews with key MoHCC informants illuminate program strengths and weaknesses.

**Results**: In start-up and year 1 (March 2013–September, 2014), ZAZIC expanded from two to 36 static VMMC sites and conducted 46,011 VMMCs; 39,840 completed from October 2013 to September 2014. From October 2014 to September 2015, 44,868 VMMCs demonstrated 13% increased productivity. In October, 2015, ZAZIC was required by its donor to consolidate service provision from 21 to 10 districts over a 3-month period. Despite this shock, 57,282 VMMCs were completed from October 2015 to September 2016 followed by 44,414 VMMCs in only 6 months, from October 2016 to March 2017. Overall, ZAZIC performed 192,575 VMMCs from March 2013 to March, 2017. The vast majority of VMMCs were completed safely by MoHCC staff with a reported moderate and severe adverse event rate of 0.3%.

**Conclusion**: The safety, flexibility, and pace of scale-up associated with the integrated VMMC model appears similar to vertical delivery with potential benefits of capacity building, sustainability and health system strengthening. These models also appear more adaptable to local contexts. Although more complicated than traditional approaches to program implementation, attention should be given to this country-led approach for its potential to spur positive health system changes, including building local ownership, capacity, and infrastructure for future public health programming.

## Background

As voluntary medical male circumcision (VMMC) safely [1–13] reduces male HIV acquisition by up to 60% [14–16], the World Health Organization (WHO) set a target of 80% VMMC coverage in 14 priority countries with high HIV burdens [17]. By the end of 2016, 14.5 million VMMCs were completed [18]. However, this still falls short of the 20 million VMMCs goal to avert an estimated 3.4 million infections and save approximately $16.5 billion in HIV-related care through 2025 [19]. Furthermore, annual gains in VMMC scale-up stagnated or decreased in eight priority countries in 2015 [20]. With projected funding reductions, programmatic innovations and efficiencies are needed to reach coverage targets.

Slower than anticipated progress in VMMC scale up may be attributed to several factors including lack of consensus on whether to implement VMMC programs as vertical (stand-alone) or horizontal (integrated) models. Currently, vertical programs predominate as they were perceived to facilitate faster service expansion during the global, rapid scale-up phase by relying on donor-dedicated funding, workers and work spaces for VMMC [21–23]. Vertical delivery systems also allow for greater control over program design, management and implementation. For one-time interventions like VMMC, some assert that stand-alone programs may be more efficient at reaching numbers during the intensive, scale-up phase to reach 80% coverage of VMMC through 2016 [24].

In contrast, integrated VMMC services may operate in multi-purpose, public service delivery points where other health services are provided by the same healthcare workers [25]. Integrated care models may provide additional clinical, public health, and efficiency benefits [26]. Integrating VMMC into routine services may help support the public sector and strengthen the overall health system [27]. As integrated programs largely rely on existing staff and infrastructure, by including VMMC as part of routine service delivery alongside other clinic services, providing VMMC services depends on improving efficiencies and rewarding productivity in the overall system. Integrated VMMC programs within the current care setting and adapted at the local level may be more easily transitioned to full local ownership by increasing long-term structures that aid sustainability [28]. Despite the potential attributes of this VMMC approach, to the authors’ knowledge, there are no published studies on its successful implementation.

In Zimbabwe, the Ministry of Health and Child Care (MoHCC) adopted a national strategy for VMMC scale-up for males ages 10–49 in 2009. Initially, vertical VMMC services were largely implemented in urban areas by one non-government organization employing a predominantly outreach model with independent, VMMC-focused teams, campaigns, and stand-alone VMMC clinics [29,30]. Early VMMC progress using the vertical model was slow [29]: by the end of 2012, Zimbabwe completed only 91,335 (4.8%) of its intended 1.9 million VMMCs [31].

**Figure 1.**
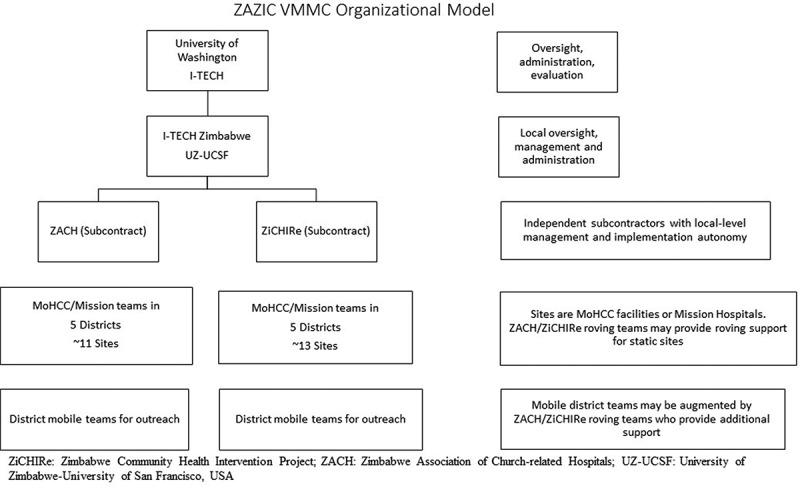
ZAZIC VMMC organizational model.

In 2013, the International Training and Education Center for Health (I-TECH) at the University of Washington, was funded by the Centers of Disease Control and Prevention, USA (CDC) through the US President’s Emergency Plan for AIDS Relief (PEPFAR) to try an additional VMMC approach: employ local partners and build health system capacity through integrated VMMC program delivery in direct partnership with the MoHCC, simultaneously promoting health system strengthening and community engagement. The ZAZIC consortium (an acronym developed with letters from each implementing partner’s name) is composed of three, local implementing partners (Figure 1). ZAZIC was initially tasked with scale-up of integrated VMMC services in 21 MoHCC- and donor-determined districts where VMMC services were previously largely unavailable; 43 other districts, including major cities, received VMMC services from the other large implementing partner.

ZAZIC conducted a process evaluation of routine program implementation to assess the program during the first 3 years of operation, aiming to understand if the program was implemented as planned and if the results can be reasonably attributed to the ZAZIC model [32]. This paper, therefore, describes the ZAZIC VMMC program approach, outputs, challenges, and achievements in the context of improving health system performance [33] and details the adaptable, community-focused program implementation in line with guidance on differentiated, HIV-related care [34]. This process evaluation may help other programs build their capacity to successfully advocate for, and overcome obstacles in a more integrated, country-led approach to VMMC service delivery.

## Methods

### Data collection

ZAZIC routine data collection, including program monitoring and evaluation (M&E) data used for this paper, comes principally from MoHCC VMMC monitoring tools, described in detail previously [35,36]. In brief, quantitative data comes from three sources: (1) the MoHCC Monthly Return Form (MRF) contains aggregated data on monthly VMMC program outputs for each site; (2) VMMC location type (static or outreach) comes from a weekly ZAZIC internal form; and (3) details on adverse events (AEs) are collected using the ZAZIC internal AE Review Tool that includes AE clinical outcomes. Additional information is outlined in the ZAZIC Standard Operating Procedure Guidelines for VMMC Program M&E (available upon request).

To complement the quantitative data, additional information on the implementation model come from data collected, but not published, in a qualitative study of the influences of the performance-based financing (PBF) incentive conducted in October 2015. The overall PBF study methodology and the broader results from the PBF-focused research were published previously [37]. In brief, for the PBF study, eight ZAZIC VMMC sites within six provinces were selected in a convenience sample to represent high- and low-performing VMMC sites: 14 key informant (KI) interviews were conducted with MoHCC administrators at the provincial, district, and clinic levels. For this process evaluation, we capitalize on unpublished data from these KI interviews that focused on VMMC program roles and overall VMMC program implementation. For this process evaluation, relevant response sections from the larger PBF study were reviewed, coded and analyzed in Atlas.ti 6.0 [38]. Thematic analysis – a flexible, realist approach to identify patterns in the data [39] – was employed to guide a process of open coding based on anticipated responses and experiences suggested by the KI interview guide. Subsequently, supplemental themes were added to both complement and provide contradictory insight [40]. An iterative process of code connections and groupings aided identification of major qualitative themes included in the results section. Results were shared with in-country teams to help ensure neutrality of the findings.

## Results

### Integrated program implementation approach

#### Build local leadership and ownership at the province, district and local levels

Within the ZAZIC consortium, Zimbabwe-based implementing partners have primary responsibility for managing program activities, thereby encouraging local ownership through our country-led approach. ZAZIC works closely with Provincial Medical Directors (PMDs) and District Medical Officers (DMOs), those primarily responsible for health services delivery, to conduct VMMCs. These provincial and district leaders are integral to the development and adaptation of the model to meet community-level needs, in accordance with guidelines for differentiated care. For example, the mix of MoHCC doctors, nurses, and other auxiliary staff involved in VMMC service delivery may differ by district or by site and over time, conforming to changing needs and priorities. As each district and site has autonomy to operate the program and deploy staff based on facility needs, staffing is highly variable by site or day. ZAZIC estimates between 180 and 540 staff across districts may be involved with VMMC program in addition to other facility duties at any time. ZAZIC supports districts and sites with VMMC roving teams to fill staffing gaps when possible. Additionally, demand creation teams are members of the communities in which they work. They mobilize their communities using demand creation strategies (messages) and activities (soccer, music, etc.) adapted for the specific communities or age groups with whom they live and work.

#### Increase local healthcare capacity

ZAZIC, in partnership with the MoHCC, implements standardized VMMC trainings in line with international best practices [41–44] including subsequent practicum on surgical and device-based VMMC for nurse and physician circumcisers (Table 1). All VMMC staff trained are employed by the MoHCC; trained staff return to their service sites within the ZAZIC districts to perform VMMCs. Nurses and clerks are also trained in routine monitoring and evaluation using MoHCC VMMC forms in line with PEPFAR guidance [45]. Demand creation training is also conducted for new and existing community mobilizers and Health Promotion Officers. To further support demand generation, additional VMMC focal persons are contracted by ZAZIC in key districts to work with the Health Promotion Officers; together they coordinate mobilizers to generate demand for outreach services, supervise outreach workers, and link interested men with transportation to improve uptake.

**Table 1.** ZAZIC training from October 2013 to March 2017.

Type of Training	Course length (days)	# Clinicians trained	# Non-clinicians trained	Total	% MC staff retention^a^
Basic VMMC Training	6 days	354	0	354	77
VMMC forceps-guided training of trainers	9 days	10	0	10	80
VMMC forceps-guided training	6 days	115	0	115	60
Nurses’ conversion course	3 days	229	0	229	75
PrePex training	5 days	319	0	319	80
PrePex training of trainers	3 days	34	0	34	62
VMMC logistics training	5 days	23	49	72	79
Dorsal slit training of trainers	9 days	25	0	25	92
Dorsal slit training	6 days	178	0	178	84
M&E data management	3 days	39	48	87	65
Emergency patient management	2 days	45	0	45	89
Rapid HIV Testing	5 days	47	0	47	100
Demand Creation	2 days	0	945	945	80
Internal Quality Assessment	2 days	6	1	7	76
Infection control (autoclave)	1 day	21	7	28	100
Total		1445	1050	2495	

^a^Training data extracted from the MoHCC TrainSmart training database, July 2017

#### Ensure VMMC integration into routine service delivery

For service delivery, routine VMMC using surgical or PrePex device-based VMMC is managed by districts as part of routine service delivery and provided free to clients. Districts have between one and five static sites with outreach performed at distant satellite health centers or diverse community settings. The MoHCC’s national supply chain program largely provides VMMC-related supplies and commodities, including VMMC kits. VMMC services and supervision are implemented predominantly by existing healthcare staff. Health facility leadership work closely with DMOs to determine site-specific VMMC service availability at static and outreach sites complemented by occasional community-based campaigns. All men who come for VMMC are strongly encouraged to test for HIV by MoHCC counseling staff in the same clinic location prior to VMMC. HIV-infected patients are linked to care. MoHCC adheres to the WHO-recommended package of VMMC services [42,46] when possible. Follow-up visits may be conducted at satellite health centers for client convenience and emergency management. All ZAZIC sites follow MoHCC AE guidelines for identification, treatment, and documentation of AEs [45]. ZAZIC, MoHCC, and CDC conduct quality assurance visits to monitor VMMC program implementation.

#### Strengthen health information

At all sites, ZAZIC uses national MoHCC forms for VMMC reporting through the district and provincial surveillance systems. During the first year as the program grew to scale, MoHCC and ZAZIC worked closely to develop and disseminate new MoHCC VMMC M&E tools which streamlined data collection to reduce redundancy and encourage data completeness. A register job aid contains clear instructions to help ensure correct form filling, including AE classification and severity. The Monthly Return Form simplifies national and donor reporting with fewer, targeted indicators that are integrated into the District Health Information System. Annual data quality audits (DQA) are conducted at a sample of ZAZIC-supported VMMC facilities to assess data quality [35], using a participatory data collection and review process with clinic staff to spur improvements to data quality.

#### Incentivize productivity through performance-based health financing

The national VMMC program adopted a $25 per VMMC performance-based financing (PBF) system [30] to offset costs, promote health system strengthening, and improve performance [47,48]. The provision of PBF, an incentive for productivity, provides valuable discretionary income for the district, facility, and staff while simultaneously requiring sites to build strong fiscal management skills and infrastructure (Table 2). ZAZIC and MoHCC monitor implementation of this funding mechanism to reduce the possibility of coercive practices. Service provision is tracked and PBF paid monthly by volume by ZAZIC to the facilities. Additional ZAZIC-specific expenditure includes VMMC training, supervision, occasional supplies, minor refurbishment, coordination, and M&E. Complementary program costs for disposable VMMC kits, site-level personnel, hospital administration, and most medical supplies are largely borne by the MoHCC or directly by donors. Previous exploration of the PBF effects found increased VMMC team motivation and improved facilities where VMMC services occur; however, the PBF also created some discord, suggesting efforts to broaden the reach of the incentives would reduce staff tension [37].

**Table 2.** MOHCC fee for service structure, 2013–2015.

Recipient	Fee in $US
Doctor/circumciser	4
Nurse (up to 3 nurses)	3
Receptionist	1
Theater assistant	1
Health promotion officer	1.50
Driver	0.50
Community nurse	1.5
Pharmacy tech	0.50
Review nurse at rural health center	1
Volunteer health workers	1
Facility fee	3
Provincial office fee	1
Total	$25

#### Demonstrate adaptability of the model: the PEPFAR pivot

In October, 2015, PEPFAR global strategy changed to focus on high HIV burden districts; in Zimbabwe, programmatic activity would focus only within the 36 sub-national units that, together, accounted for 80% of people living with HIV/AIDS [49]. Therefore, ZAZIC’s VMMC efforts were reduced from 21 to only 10 high HIV burden districts, decreasing from 36 to 18 static sites. As productivity targets remained the same, each of the 10 priority districts had dramatic increases in expected VMMC outputs. With the dual goals of integration and swift acceleration in 10 remaining districts, the integrated model was stretched. ZAZIC responded rapidly through several programmatic steps. First, clear communication was sent to non-priority sites on funding end date; MoHCC aided this effort. Second, ZAZIC recognized that the increased productivity targets would outstrip the capacity of local institutions to meet the targets alone. Therefore, ZAZIC transformed operations into a *blended* implementation model that employed additional non-MoHCC mobile and outreach staff to support MoHCC staff performing VMMC in a variety of locations, and enabling extended or weekend hours for VMMC service delivery. Third, VMMC trainings for additional personnel were expanded to increase the number of trained VMMC providers in priority districts. Fourth, ZAZIC expanded the reach of VMMC services by adding more outreach sites and reassigning vehicles from non-priority to priority districts, maintaining the proportion of VMMCs occurring in outreach sites at approximately 85%. Last, demand creation efforts were prioritized in rural areas, focusing on community-based entertainment activities such as soccer tournaments and music galas with a VMMC focus. New payment modalities for mobilization efforts were developed to incentivize mobilizers. Non-priority districts were not eligible for PBF payments and largely did not maintain VMMC services.

### Program outputs

**Figure 2.**
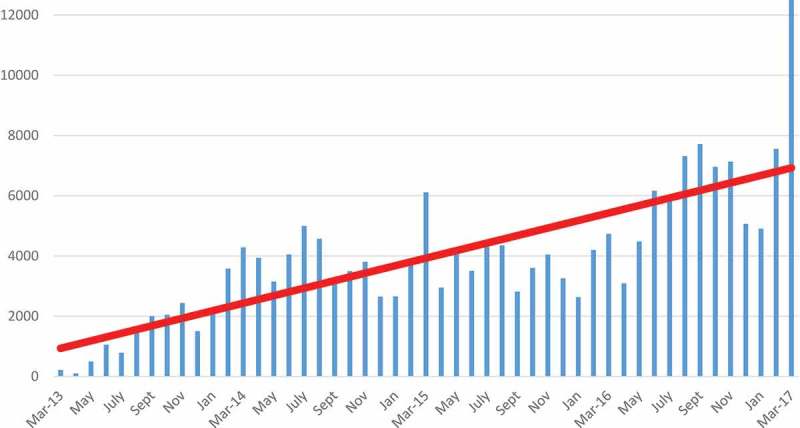
Number of MCs conducted by ZAZIC across all sites by month, March 2013–March 2017.

During the integrated implementation phase, from March, 2013 through September, 2014, ZAZIC expanded from 2 to 36 static VMMC sites and conducted 46,011 VMMCs: 39,840 (including 1085 using the non-surgical PrePex device) were conducted from October 2013 to September 2014 (FY14). Over FY14, ZAZIC completed an average of 2654 and 3985 VMMCs/month in the first and second 6-month period, respectively, an increase of 50%. Subsequently, in FY15, an additional 44,868 VMMCs were performed (including 3452 PrePex), demonstrating a 13% increase in annual VMMC productivity. In the blended period, from October 2015–September 2016, productivity increased further with 57,282 VMMCs (including 12,652 PrePex (22%)), reflecting an overall 27.7% growth over the previous 12-month period with VMMCs performed at approximately 18 static sites. ZAZIC ramped up again from October 2016 to March 2017 and conducted 44,414 VMMCs in just 6 months by increasing the static sites to 24, adding additional demand creation officers and focused community engagement using soccer tournaments and music galas. This achievement exceeding higher donor targets and marked ZAZIC’s most productive 6-month period. Overall, ZAZIC safely performed 192,575 VMMCs from March 2013–March, 2017 during its first 3 years of operation (Figure 2 and Table 3).

**Table 3.** Moderate and severe adverse events and follow-up by month, October 2013 to September 2016.

	2013						2014						
	October	November	December	January	February	March	April	May	June	July	August	September	FY14 Total
AEs	11	3	4	11	21	23	30	12	46	17	13	2	193
AE rate %	0.59	0.12	0.26	0.65	0.64	0.56	0.83	0.38	1.18	0.36	0.28	0.06	0.49
Total MCs	2047	2440	1505	2068	3579	4290	3937	3153	4049	4997	4568	3207	39,840
	2014						2015						FY15 Total
AEs	11	9	5	7	22	32	15	14	17	7	9	9	157
AE rate %	0.32	0.24	0.18	0.28	0.60	0.56	0.47	0.34	0.50	0.16	0.21	0.31	0.36
Total MCs	3496	3807	2653	2657	3879	6110	2952	4133	3505	4503	4352	2821	44,868
	2015						2016						
AEs	6	7	11	4	9	39	14	22	15	28	20	8	183
AE rate %	0.17	0.17	0.34	0.15	0.22	0.82	0.45	0.49	0.24	0.50	0.27	0.10	0.33
Total MCs	3604	4047	3257	2634	4199	4737	3095	4485	6167	6021	7319	7717	57,282
	2016						2017						
AEs	8	13	3	0	11	13							48
AE rate %	0.11	0.18	0.06	0.00	0.15	0.01							0.10
Total MCs	6957	7131	5068	4910	7559	12,789							44,414

Safety of VMMC clients was assured during this period of scale-up and transition with reported rates of adverse events (AE) remaining under 0.5% over the first 3 years (Table 3) [36].

### Implementation strengths and weaknesses

The 14 MoHCC key informant (KI) interviews included: 1 Senior Health Promotion Officer; 2 Matrons; 5 Provincial Medical Directors (PMD); 4 Medical Superintendents; and 2 District Medical Officers (DMO). Results highlighted several strengths and challenges of the integrated implementation model.

#### ZAZIC model strengths

First, it appears clear that local MoHCC staff feel ownership over the program. When asked their roles, responses were similar to this PMD who noted that his role was ‘the overall in-charge of all the programs in the district and one of them being the Voluntary Medical Male Circumcision.’ KIs were also likely to mention their primary roles in the entire VMMC process.

*[VMMC] is a Ministry of Health program and in our case as local authority we have to toll the policy of the central government. The ministry took the program on board, and we also have to take the program on board. We have to create demand for the program and also create awareness in the community to increase the uptake of the program.*

Another KI noted his authority and ownership of the VMMC program more bluntly:

*Our partners think they are overall responsible for all the activities because they have got money, but we think that we should be overall responsible. We should decide how the program runs.*

Second, it seems that ZAZIC’s model is integrated into routine service delivery. KIs generally noted that, ‘integration of the program is not a problem. We will do as we normally do with other programs.’ The linkages with other HIV-related services is also evident as men found HIV-positive through testing as part of VMMC, ‘they can be easily referred and commenced on ART.’ Several KIs also noted similar directives to this respondent:

*The VMMC program is part of the Ministry of Health program so we make sure that everyone understands that [VMMC] it is part of hospital business…This means that we have integrated circumcision to our health services*.

Last, MoHCC staff benefit from the VMMC trainings. One KI explained that staff VMMC training ‘is an additional skill and qualification’ while another mentioned that a program ‘advantage is that a number of staff has trained in this [VMMC] skill which is a lifetime skill.’ Others may believe similarly to this KI who noted the reach of program training and involvement:

*[The VMMC program] it’s working very well, I think the team is working very well. There is lots of team work. We have many nurses who are trained in VMMC. It’s more of team work at the hospital because we have the nurses, clerks, administrators, and quite a number of people in the hospital involved.*

#### ZAZIC program challenges

KIs also provided several key insights into program challenges. First, several KIs suggested that further synergies between VMMC and other health interventions could be advantageous to demonstrate full integration of VMMC into routine service delivery. Recommendations included rolling VMMC messages into scheduled health education talks and encouraging providers in other clinic service areas to actively refer eligible men to VMMC as part of standard practice. Others suggested similarly to this KI:

*[VMMC] can be integrated with HIV testing and counseling since HIV testing and counseling is the main entry point to circumcision... Also circumcision is an HIV preventive measure. We could also take advantage of the STI clinics to talk about circumcision.*

Moreover, additional improvements in client access to VMMC were noted. One KI recommended that VMMC access should be available at all lower-level, static healthcare clinics, since that would demonstrate ‘*that we have done the job since anyone can access [VMMC] at anytime*.’ A number of other KIs also mentioned VMMC decentralization efforts to extend routine service availability to more lower-level facilities.

*The biggest challenge is that this VMMC thing is outreach based. We want it to become part of the day to day business of every facility. If a nurse can do it from the hospital and go for outreach why can’t a nurse at that outreach clinic do it on a daily basis?*

Last, although KIs believed that the VMMC program strengthened staff skills, the related issues of worker attrition and staff movement reduced the effects of capacity building. To sustain a more full and consistent integration of VMMC services, several KIs noted the need for continuous opportunities for VMMC trainings to compensate for staff turnover. Additional trainings could also extend access by ensuring VMMC capacity at more rural static sites.

*There is staff attrition, a lot of movements among health personnel. You find that all trained doctors for VMMC…have gone so we need to re-train other doctors*.

## Discussion

This process evaluation helps demonstrate that ZAZIC largely implemented its model as planned and suggests that ZAZIC results may be attributable to its integrated and blended implementation model. ZAZIC successfully and safely conducted 192,575 VMMCs during its first 3 years of implementation. ZAZIC weathered an intense shock to the integrated model, resulting in a successful transition to a more blended program approach, demonstrating immense flexibility and adaptability. During implementation, ZAZIC also partnered with local government leaders to support service delivery with periodic community-adapted campaigns, demonstrating a collaborative, contextual approach to implementation in line with guidance for HIV-related differentiated care. Employing this model, overall ZAZIC performance these first years appears similar to early VMMC productivity from other predominantly vertical programs including Kenya, Tanzania, and South Africa [24,50] and appears to exceed output in early program years of other predominantly vertical programs in Botswana, Malawi, and Mozambique [18,51]. Within Zimbabwe, the ZAZIC approach outpaces earlier achievements by the vertical model [30,31].

Vertical VMMC programs are often justified to achieve results quickly; however, ZAZIC shows that a more sustainable, integrated (or blended) model may achieve similarly. According to routine M&E data, ZAZIC’s recent achievements continue to demonstrate excellent progress towards MoHCC targets: by March, 2017, ZAZIC reached 90% of its semi-annual target as compared to 56% of the semi-annual target achieved by the other large-scale partner organization in Zimbabwe [52]. ZAZIC also implemented VMMC safely [36] with reported AE rates lower than the global safety standard of 2% in all 3 years of operation [53–55]. While more logistically challenging than the predominantly vertical approach, and leaders note some weaknesses in program synergies, client access, and VMMC staff training, the progress evidenced by ZAZIC suggests that an integrated or blended approach may positively affect VMMC short-term outputs and supports continued efforts at integration of programs to strengthen local health systems [56].

Among the greatest accomplishments of the integrated and blended approach is that district teams independently completed approximately 85% of VMMCs due, in large part, to broad support from the district and provincial health leadership. Although additional mobile teams using non-MoHCC staff contributed to the success of the transition to only 10 districts, MoHCC appears to demonstrate program ownership and improved sustainability for the future. Demand creation adapted to local community engagement complemented this approach. Moreover, key VMMC indicators were incorporated into the national District Health Information System, showing integration of this program into routine service delivery and reporting requirements. Also, in 2014, the MoHCC adopted a task shifting approach to promote nurse-led VMMC for both surgical and device-based services, improving provider coverage for integrated VMMC activities. From DQA implementation and reported AEs, it appears ZAZIC oversight in combination with district-based implementation helped ensure progress towards greater protocol adherence, infection prevention, quality improvement, and compliance with safety standards in a manner that encourages sustainability of these efforts [35].

Moreover, for routine service delivery, the integrated and blended model’s heavy reliance on the existing facility, staff, commodity and supply infrastructure, supplemented by PBF, appears to help reduce donor costs and incentivizes providers to incorporate VMMC into their schedules. The integrated model also relies on cost sharing: the healthcare workers who perform VMMCs and supportive services, facilities for service delivery, and logistics systems for supplies are provided by the MoHCC. Management and supervision, after start-up, are a stable ZAZIC investment to promote quality service delivery. Last, the PBF component, supported by donors and MoHCC, and managed by ZAZIC, encourages productive service delivery from local clinic teams as part of routine duties. Keeping healthcare workers in their workplaces potentially reduces disruption to district healthcare staffing or decreased clinic productivity.

ZAZIC success is not without some formidable challenges, some due to its integrated model. First, we had a slower pace of scale-up than might be anticipated in a vertical approach due to the need for local buy-in, ownership, and intensive participation. The first 9 months of critical partnership building reflect this slower start-up period. Second, with the integrated model, physicians and nurses have multiple competing demands and equipment may be shared across departments. VMMC capacity is growing; however, staff training achievements are challenged by attrition and staff mobility. Reliance on practicing providers is also less well suited to the campaign approach that may generate large numbers in a short time period. Also, the integrated model largely relies on the existing health system for strategic information; revisions in the national VMMC reporting tools were needed to improve initial data quality and aggregation flow [30]. For both vertical and horizontal models, creating demand for VMMC remains the key to success. However, national and lower-level demand creation strategies still require additional funding and effort to better reach more dispersed, rural populations. Last, similar to vertical programs, external support remains critical for further decentralization and integration success during this expansion phase. Despite these challenges and trade-offs, the results from this integrated and blended approach demonstrate ZAZIC’s ability to overcome obstacles and meet both safety and productivity expectations with the potential added benefit of being sustained beyond intensive funding.

### Limitations

We qualitatively compare our integrated and blended program productivity to that of other more vertical VMMC programs in Zimbabwe and in the region, suggesting the success of this innovative model. We do not detail program implementation of a vertical model. More rigorous quantitative comparison between integrated and vertical programs was not undertaken as part of this process evaluation of routine program implementation. As an implementing partner working at scale, we are restricted from engaging in formal research comparing models. Moreover, the qualitative data used for this study was collected during the integrated, but not blended, implementation period. Furthermore, although we report on the use of the PBF, analysis of VMMC program costs are outside the scope of this paper. Despite these limitations, this process evaluation of the ZAZIC VMMC program and its results is the first to provide insight into this innovative program model.

## Conclusion

This process evaluation shows that ZAZIC’s integrated, blended approach to VMMC demonstrates a productive pace of scale-up and client safety while likely encouraging simultaneous health system strengthening. Although local leaders note that improvements could be made in program reach, additional staff training, and decentralization efforts, the model appears to work well to leverage and augment existing VMMC capacity across multiple sites in 21 districts. ZAZIC’s locally led consortium also successfully transitioned its broader program to meet changing PEPFAR priorities by focusing in depth on 10 priority districts. Throughout the transition, ZAZIC continued to safely improve productivity, further suggesting advantages of the ZAZIC approach. ZAZIC also sets an example of a successful MoHCC partnership with MoHCC receiving implementation support and gaining VMMC capacity through this joint, country-led implementation. As recent HIV combination prevention trials highlighted the importance of community-based adaptations to extend the reach of public health programs [57], this locally led model further merits consideration. ZAZIC’s integrated, blended model shows potential for empowering local communities to safely implement successful VMMC programs.

In the future, ZAZIC aims to evaluate the program more rigorously through both qualitative and quantitative methods. Greater understanding of the implications of transition from integration to blended implementation on VMMC service delivery, including the effects on other routine services within health facilities where VMMCs are performed, would provide insight into the model’s impact. Moreover, as additional VMMC roving teams were added to meet targets, assessment of the effects of these supplemental teams on site engagement and VMMC program sustainability, overall, would be informative. Furthermore, considering the continued HIV epidemic and the fiscal challenges of PEPFAR, PEPFAR should facilitate more rigorous, independent, qualitative and quantitative research comparing vertical versus integrated/blended programs with respect to acceptability, performance and sustainability. Continued consideration for VMMC program integration efforts and support for blended delivery models, in the near and long terms, are warranted to reinforce commitments to VMMC targets and to support health systems in Zimbabwe and the region.

## Paper context

Voluntary medical male circumcision (VMMC) expansion falls short of targets. Emphasis on vertical (stand-alone) implementation models may contribute to slower scale-up. In contrast, ZAZIC’s integrated VMMC approach in Zimbabwe primarily uses Ministry of Health workers and infrastructure. In 3 years, ZAZIC safely performed 192,575 VMMCs with low reported moderate and severe adverse events (0.3%). ZAZIC productivity appears similar to vertical delivery with additional advantages in local ownership, program adaptability, capacity building, and health system strengthening.

## References

[1] Reed J, Grund J, Liu Y, et al Evaluation of loss-to-follow-up and post-operative adverse events in a voluntary medical male circumcision program in Nyanza Province, Kenya. J Acquir Immune Defic Syndr. 2015;69(1):e13–23. 10.1097/QAI.0000000000000535 25942466

[2] Lebina L, Taruberekera N, Milovanovic M, et al Piloting PrePex for adult and adolescent male circumcision in South Africa – pain is an issue. PLoS One. 2015;10:e0138755. 26405786 10.1371/journal.pone.0138755 PMC4583405

[3] Phili R, Abdool-Karim Q, Ngesa O. Low adverse event rates following voluntary medical male circumcision in a high HIV disease burden public sector prevention programme in South Africa. J Int AIDS Soc. 2014;17. 10.7448/IAS.17.1.19275 PMC4236629 25406951

[4] Kigozi G, Musoke R, Kighoma N, et al Safety of medical male circumcision in human immunodeficiency virus-infected men in Rakai, Uganda. Urology. 2014;83:294–12. 24286598 10.1016/j.urology.2013.08.038

[5] Feldblum PJ, Odoyo-June E, Obiero W, et al Safety, effectiveness and acceptability of the PrePex device for adult male circumcision in Kenya. PLoS One. 2014;9:e95357. 24788898 10.1371/journal.pone.0095357 PMC4006910

[6] Ashengo TA, Grund J, Mhlanga M, et al Feasibility and validity of telephone triage for adverse events during a voluntary medical male circumcision campaign in Swaziland. BMC Public Health. 2014;14:858. 25134856 10.1186/1471-2458-14-858 PMC4150954

[7] Duffy K, Galukande M, Wooding N, et al Reach and cost-effectiveness of the PrePex device for safe male circumcision in Uganda. PLoS One. 2013;8:e63134. 23717402 10.1371/journal.pone.0063134 PMC3661578

[8] World Health Organization WHO technical advisory group on innovations in male circumcision: evaluation of two adult devices. Geneva: WHO; 2013.

[9] Musiige AM, Ashengo TA, Stolarsky G, et al Participant experiences and views of odor and PrePex device removal pain in a VMMC pilot study in Botswana. J Acquir Immune Defic Syndr. 2016;72:S73. 27331595 10.1097/QAI.0000000000000765 PMC4936428

[10] Bitega JP, Ngeruka ML, Hategekimana T, et al Safety and efficacy of the PrePex device for rapid scale-up of male circumcision for HIV prevention in resource-limited settings. J Acquir Immune Defic Syndr. 2011;58:e127–e134. 21909032 10.1097/QAI.0b013e3182354e65

[11] Kigozi G, Musoke R, Watya S, et al The safety and acceptance of the PrePex device for non-surgical adult male circumcision in Rakai, Uganda. A non-randomized observational study. PLoS One. 2014;9:e100008. 25144194 10.1371/journal.pone.0100008 PMC4140666

[12] Mutabazi V, Kaplan SA, Rwamasirabo E, et al One-arm, open-label, prospective, cohort field study to assess the safety and efficacy of the PrePex device for scale-up of nonsurgical circumcision when performed by nurses in resource-limited settings for HIV prevention. J Acquir Immune Defic Syndr. 2013;63:315–322. 23466648 10.1097/QAI.0b013e31828e6412

[13] Galukande M, Duffy K, Bitega JP, et al Adverse events profile of PrePex a non-surgical device for adult male circumcision in a Ugandan urban setting. PLoS One. 2014;9:e86631. 24489754 10.1371/journal.pone.0086631 PMC3904949

[14] Gray RH, Kigozi G, Serwadda D, et al Male circumcision for HIV prevention in men in Rakai, Uganda: a randomised trial. Lancet. 2007;369:657–666. 17321311 10.1016/S0140-6736(07)60313-4

[15] Bailey RC, Moses S, Parker CB, et al Male circumcision for HIV prevention in young men in Kisumu, Kenya: a randomised controlled trial. Lancet. 2007;369:643–656. 17321310 10.1016/S0140-6736(07)60312-2

[16] Auvert B, Taljaard D, Lagarde E, et al Randomized, controlled intervention trial of male circumcision for reduction of HIV infection risk: the ANRS 1265 trial. PLos Med. 2005;2:e298. 16231970 10.1371/journal.pmed.0020298 PMC1262556

[17] WHO U Joint strategic action framework to accelerate the scale-up of voluntary medical male circumcision for HIV prevention in eastern and southern Africa (2012–2016). Geneva: UNAIDS; 2011.

[18] World Health Organization Voluntary medical male circumcision for HIV prevention in 14 priority countries in eastern and southern Africa. Geneva: WHO; 2017.

[19] Njeuhmeli E, Forsythe S, Reed J, et al Voluntary medical male circumcision: modeling the impact and cost of expanding male circumcision for HIV prevention in eastern and southern Africa. PLoS Med. 2011;8:e1001132. 22140367 10.1371/journal.pmed.1001132 PMC3226464

[20] World Health Organization Voluntary medical male circumcision for HIV prevention in 14 priority countries in east and southern Africa. Geneva: WHO; 2016 [cited 2017 328]. Available from: http://apps.who.int/iris/bitstream/10665/246174/1/WHO-HIV-2016.14-eng.pdf

[21] Lissouba P, Taljaard D, Rech D, et al A model for the roll-out of comprehensive adult male circumcision services in African low-income settings of high HIV incidence: the ANRS 12126 Bophelo Pele Project. PLoS Med. 2010;7:e1000309. 20652013 10.1371/journal.pmed.1000309 PMC2907271

[22] Mwandi Z, Murphy A, Reed J, et al Voluntary medical male circumcision: translating research into the rapid expansion of services in Kenya, 2008–2011. PLoS Med. 2011;8:e1001130. 22140365 10.1371/journal.pmed.1001130 PMC3226459

[23] Mahler HR, Kileo B, Curran K, et al Voluntary medical male circumcision: matching demand and supply with quality and efficiency in a high-volume campaign in Iringa Region, Tanzania. PLoS Med. 2011;8:e1001131. 22140366 10.1371/journal.pmed.1001131 PMC3226544

[24] Reed JB, Njeuhmeli E, Thomas AG, et al Voluntary medical male circumcision: an HIV prevention priority for PEPFAR. J Acquir Immune Defic Syndr. 2012;60:S88. 22797745 10.1097/QAI.0b013e31825cac4e PMC3663585

[25] World Health Organization Integrated health services – what and why? Geneva: WHO; 2008.

[26] Sweeney S, Obure CD, Maier CB, et al Costs and efficiency of integrating HIV/AIDS services with other health services: a systematic review of evidence and experience. Sex Transm Infect. 2011;sextrans-2011. 10.1136/sextrans-2011-050199 22158934

[27] Buvé A, Delvaux T, Criel B Delivery of male circumcision services: “Festina lente”. Reprod Health Matters. 2007;15:57–61. 17512376 10.1016/S0968-8080(07)29305-X

[28] Auvert B, Marseille E, Korenromp EL, et al Estimating the resources needed and savings anticipated from roll-out of adult male circumcision in sub-Saharan Africa. PLoS One. 2008;3:e2679. 18682725 10.1371/journal.pone.0002679 PMC2475667

[29] Ashengo TA, Hatzold K, Mahler H, et al Voluntary medical male circumcision (VMMC) in Tanzania and Zimbabwe: service delivery intensity and modality and their influence on the age of clients. PloS One. 2014;9:e83642. 24801882 10.1371/journal.pone.0083642 PMC4011872

[30] Ministry of Health and Child Care Zimbabwe Accelerated strategic and operational plan 2014–2018: voluntary medical male circumcision. Harare, Zimbabwe: Ministry of Health and Child Care; 2014.

[31] World Health Organization Regional Office for Africa Progress in scaling up voluntary medical male circumcision or HIV prevention in east and southern Africa January–December 2012. 2013 [cited 2014 1027]. Available from: http://www.malecircumcision.org/country_updates/documents/Progress%20in%20scaling%20up%20VMMC_Dec2013.pdf

[32] Linnan L, Steckler A Process evaluation for public health interventions and research. San Francisco (CA): Jossey-Bass; 2002.

[33] World Health Organization Everybody’s business–strengthening health systems to improve health outcomes: WHO’s framework for action. Geneva, Switzerland: World Health Organization; 2007.

[34] International AIDS Society (IAS). Differentiated care for HIV: a decision framework for antiretroviral therapy. Durban, South Africa. July, 2016. [cited 18 October 2017]. Available from: http://www.differentiatedcare.org/Portals/0/adam/Content/yS6M-GKB5EWs_uTBHk1C1Q/File/Decision%20Framework.pdf

[35] Xiao Y, Bochner A, Makunike B, et al Challenges in data quality: the influence of data quality assessments on data availability and completeness in a voluntary medical male circumcision programme in Zimbabwe. BMJ Open. 2017;7:e013562. 10.1136/bmjopen-2016-013562 PMC5278271 28132009

[36] Bochner A, Feldacker C, Makunike B, et al Adverse event profile of a mature voluntary medical male circumcision progra mme performing PrePex and surgical procedures in Zimbabwe. J Int AIDS Soc. 2017;20:21394. 10.7448/IAS.20.1.21394 PMC5467584 28362066

[37] Feldacker C, Bochner AF, Herman-Roloff A, et al Is it all about the money? A qualitative exploration of the effects of performance-based financial incentives on Zimbabwe’s voluntary male medical circumcision program. PloS One. 2017;12:e0174047. 28301588 10.1371/journal.pone.0174047 PMC5354455

[38] Muhr T ATLAS. ti 6.0 [version 6]. Berlin: ATLAS ti Scientific Software Development GmbH; 2004.

[39] Braun V, Clarke V Using thematic analysis in psychology. Qual Res Psychol. 2006;3:77–101.

[40] Glaser BG Basics of grounded theory analysis: emergence vs forcing. Mill Valley, CA: Sociology Press; 1992.

[41] President’s Emergency Plan for AIDS Relief PEPFAR’s best practices for voluntary medical male circumcision site operations: a service guide for site operations. 2013 [cited 2014 5]. Available from: http://www.malecircumcision.org/resources/documents/VMMC%20Best%20Practices03.04.2013_web.pdf

[42] World Health Organization Manual for male circumcision under local anaesthesia. Geneva: WHO; 2009.

[43] Population Services International CoSoE, Central and Southern Africa (COSECSA), U.S. Centers for Disease Control and Prevention Adverse event action guide for voluntary medical male circumcision by surgery or device. 2016 [cited 2017 December 16]. Available from: https://www.malecircumcision.org/resource/adverse-event-action-guide-voluntary-medical-male-circumcision-surgery-or-device-2nd.

[44] World Health Organization Guideline on the use of devices for adult male circumcision for HIV prevention. 2013 [cited 2017 32]. Available from: http://apps.who.int/iris/bitstream/10665/93178/1/9789241506267_eng.pdf?ua=1 24624479

[45] President’s Emergency Plan for AIDS Relief PEPFAR monitoring, evaluation, and reporting indicator reference guide. March 2015 ed Washington (DC): PEPFAR; 2015.

[46] World Health Organization Considerations for implementing models for optimizing the volume and efficiency of male circumcision services. 2010 [cited 2014 61]. Field testing edition: February 2010. Available from: http://www.malecircumcision.org/programs/documents/mc_MOVE_2010_web.pdf

[47] Petersen LA, Woodard LD, Urech T, et al Does pay-for-performance improve the quality of health care? Ann Intern Med. 2006;145:265–272. 16908917 10.7326/0003-4819-145-4-200608150-00006

[48] Low-Beer D, Afkhami H, Komatsu R, et al Making performance-based funding work for health. PLoS Med. 2007;4:e219. 17713979 10.1371/journal.pmed.0040219 PMC1949844

[49] Relief PsEPfA PEPFAR Zimbabwe Country Operational Plan (COP) 2016 strategic direction summary. 2016 [cited 2017 815]. Available from: https://www.pepfar.gov/documents/organization/257623.pdf

[50] Bertrand JT, Rech D, Omondi Aduda D, et al Systematic monitoring of voluntary medical male circumcision scale-up: adoption of efficiency elements in Kenya, South Africa, Tanzania, and Zimbabwe. PLoS One. 2014;9:e82518. 24801374 10.1371/journal.pone.0082518 PMC4011576

[51] World Health Organization WHO progress brief: voluntary medical male circumcision for HIV prevention in 14 priority countries in east and southern Africa. 2015 [cited 2016 31]. Available from: http://apps.who.int/iris/bitstream/10665/179933/1/WHO_HIV_2015.21_eng.pdf?ua=1&ua=1

[52] Murenje V, Maponga B PEPFAR supported VMMC program performance review. Oct ‘16 to Mar ‘17, PSI Zimbabwe and ZAZIC [Powerpoint presentation]. Forthcoming; 2017.

[53] World Health Organization WHO technical advisory group on innovations in male circumcision: evaluation of two adult devices: meeting report. Geneva: WHO; 2013.

[54] Byabagambi J, Kigonya A, Lawino A, et al A guide to improving the quality of safe male circumcision in Uganda. 2015 [cited 2016 812]. Available from: https://www.usaidassist.org/sites/assist/files/uganda_guide_to_improving_the_quality_of_smc_a4_feb2015_ada.pdf

[55] Zimbabwe Ministry of Health and Child Care Accelerated strategic and operational plan 2014–2018. Harare, Zimbabwe: Ministry of Health and Child Care; 2014.

[56] Buckley GJ, Lange JE, Peterson EA Investing in global health systems: sustaining gains, transforming lives. National Academies Press; 2014. 25299035

[57] President’s Emergency Plan for AIDS Relief PEPFAR Country/Regional Operational Plan (COP/ROP) 2017 guidance: DRAFT – December 30, 2016. Washington (DC): PEPFAR; 2016.

